# Effects of E2HSA, a Long-Acting Glucagon Like Peptide-1 Receptor Agonist, on Glycemic Control and Beta Cell Function in Spontaneous Diabetic db/db Mice

**DOI:** 10.1155/2015/817839

**Published:** 2015-08-17

**Authors:** Shaocong Hou, Caina Li, Yi Huan, Shuainan Liu, Quan Liu, Sujuan Sun, Qian Jiang, Chunming Jia, Zhufang Shen

**Affiliations:** State Key Laboratory of Bioactive Substances and Functions of Natural Medicines, Institute of Materia Medica, Chinese Academy of Medical Sciences and Peking Union Medical College, Beijing 100050, China

## Abstract

Glucagon like peptide-1 (GLP-1) receptor agonists such as exendin-4 have been widely used but their short half-life limits their therapeutic value. The recombinant protein, E2HSA, is a novel, long-acting GLP-1 receptor agonist generated by the fusion of exendin-4 with human serum albumin. In mouse pancreatic NIT-1 cells, E2HSA activated GLP-1 receptor with similar efficacy as exendin-4. After single-dose administration in ICR mice, E2HSA showed prolonged glucose lowering effects which lasted up to four days and extended inhibition on gastric emptying for at least 72 hours. Chronic E2HSA treatment in db/db mice significantly improved glucose tolerance, reduced elevated nonfasting and fasting plasma glucose levels, and also decreased HbA1c levels. E2HSA also increased insulin secretion and decreased body weight and appetite. Furthermore, immunofluorescence analysis showed that E2HSA increased *β*-cell area, improved islet morphology, and reduced *β*-cell apoptosis. In accordance with the promotion of *β*-cell function and survival, E2HSA upregulated genes such as Irs2, Pdx-1, Nkx6.1, and MafA and downregulated the expression levels of FoxO1 and proapoptotic Bcl-2 family proteins. In conclusion, with prolonged glucose lowering effects and promoting *β*-cell function and survival, the fusion protein, E2HSA, is a promising new therapeutic for once weekly treatment of type 2 diabetes.

## 1. Introduction

Type 2 diabetes mellitus (T2DM) has two major characteristics: reduced insulin sensitivity linked to obesity and impaired insulin secretion due to *β*-cell dysfunction [1]. *β*-cell dysfunction occurs when the demand for insulin finally overwhelms the capacity of the *β*-cell to respond [2], which results in severely reduced insulin secretion [3] and ultimately *β*-cell death [4]. Few available drugs can enhance *β*-cell viability and restore their ability to synthesize and secrete insulin without side effects such as hypoglycemia or weight gain. Glucagon like peptide-1 (GLP-1) represents such a therapeutic target to offer clinical benefits without the undesirable side effects mentioned above. GLP-1 is an incretin hormone secreted by enteroendocrine intestine L cells and plays an important role in glucose homeostasis and nutrient metabolism [5]. The action of GLP-1 is initiated by its binding to the GLP-1 receptor [6], which is expressed in islet *α*-cells and *β*-cells and in other tissues, including the central and peripheral nervous systems and gastrointestinal tract. After binding to GLP-1 receptor, GLP-1 stimulates insulin secretion in a glucose dependent manner, which means there is no or little risk of hypoglycemia [7]. Other effects include suppression of glucagon secretion, delayed gastric emptying, and increased satiety [6]. Through multiple signal transduction pathways, GLP-1 also promotes the proliferation and neogenesis of islet *β*-cells while at the same time reducing their apoptosis [8].

Native, endogenous GLP-1 is rapidly degraded by dipeptidyl peptidase-4 (DPP-4) [9], resulting in a half-life of only 1-2 minutes [10]. GLP-1 can be stabilized against DPP-4 through substituting some amino acids, but elimination by the kidneys still renders it short-lived (half-life about 4-5 min) [11]. Therefore, more and more research is focusing on DPP-4-resistant, long-acting GLP-1 receptor (GLP-1R) agonists. Exendin-4, a peptide originally isolated from lizard venom [12], shares a 53% amino acid sequence with mammalian GLP-1 and is resistant to DPP-4-mediated degradation [13]. Its synthetic form, exenatide, was the first GLP-1R agonist available on the market. Exenatide has a relatively longer circulating half-life of 90–216 minutes (average 2.4 h) and thus needs to be injected twice daily [14]. The second available GLP-1R agonist, liraglutide, extends its half-life by noncovalently interacting with albumin, but due to clearance by the kidney [15] once daily administration is still necessary [16].

Although exenatide and liraglutide have beneficial effects on blood glucose control, the requirement for once or twice daily injections limits their clinical use. Because the kidney generally filters out molecules below 60 kDa [17] and albumin (~67 kDa) has a much longer half-life in humans [18] a fused exendin-4-albumin protein should exhibit a prolonged circulating half-life. E2HSA, the recombinant protein we report here, is the product of such a strategy. E2HSA is a recombinant exendin-4-human serum albumin (HSA) fusion protein which retains the GLP-1 receptor binding activity of exendin-4 and as such is expected to exert glucose lowering effects with a prolonged duration. Thus, the aims of the present study were to evaluate its acting time and its antidiabetic efficacy in vivo. The effect on *β*-cell function and survival after chronic treatment was also assessed to elucidate possible mechanisms.

## 2. Materials and Methods

### 2.1. Materials

E2HSA (Patent no. CN101525386A) was provided by Zhejiang Huayang Pharma Inc. (China) as freeze-dried powder. Exendin-4 (exenatide) was a product of Eli Lilly and Company (USA). Lipofectamine 2000 was obtained from Invitrogen (USA). Rat anti-insulin antibody was purchased from R&D Inc. (USA). Rabbit anti-glucagon antibody, rabbit anti-FoxO1 antibody, rabbit anti-phospho-FoxO1 antibody, rabbit anti-BAD antibody, rabbit anti-phospho-BAD antibody, rabbit anti-Bim antibody, rabbit anti-Bcl-2 antibody, rabbit anti-Bcl-XL antibody, and rabbit anti-Phospho-Erk1/2 antibody were all purchased from Cell Signaling Technology Inc. (USA). The in situ cell death detection kit was a product of Roche Inc. (USA).

### 2.2. Animals

Male ICR mice weighing 20–22 g were purchased from Vital River Laboratory Animal Technology Co. Ltd. (Beijing, China) and housed five per cage with access to standard chow (Research Diets, Inc., Beijing, China) and water at constant room temperature (22 ± 3°C) in a 12 h light/dark cycle.

Female db/db (BKS.Cg-*m*+/+*Lepr* ^db^/J) mice aged 6-7 weeks were purchased from Changzhou Cavens Laboratory Animal Co. Ltd. (Changzhou, China). db/db mice were housed four or three per cage at constant room temperature (22 ± 3°C) in a 12 h light/dark cycle and fed ad libitum with a special diet (protein content is higher; other ingredients are the same as standard diet) supplied by Changzhou Cavens Laboratory Animal Co. Ltd. (Changzhou, China). Age-matched heterozygotes db/m mice were used as control animals. db/m mice were housed five per cage under the same conditions and fed ad libitum with standard diet (Research Diets, Inc., Beijing, China).

All animals were handled in accordance with the standards for laboratory animals established by China (GB14925-2001), and all efforts were made to minimize suffering.

### 2.3. GLP-1 Receptor Activation In Vitro

Mouse pancreatic *β*-cell line NIT-1 was purchased from ATCC. Cells were cultured with DMEM/F12 media containing 10% (v/v) fetal bovine serum and 100 mg/L penicillin-streptomycin and incubated at 37°C in 5% CO_2_ atmosphere. The plasmid Peak12 RIP-CRE 6x Luciferase was constructed as described [19]. Briefly, six copies of a GLP-1 specific cAMP-response element-like sequence of rat insulin promoter (RIP-CRE) were inserted in Peak12 Sx Syn E-Luciferase plasmid upstream of the Luciferase reporter gene. After the plasmid was transfected into NIT-1 cells, Luciferase expression can be specifically and dose-dependently induced by GLP-1 receptor agonists via GLP-1 receptor activation.

Cells were seeded in 6-well plates and transiently transfected with Peak12 RIP-CRE 6x Luciferase plasmid using Lipofectamine 2000 following the manufacture's protocols. Twenty hours later, the cells were transferred to 96-well plates and treated with indicated concentrations of E2HSA and exendin-4 for 24 hours. Cells were harvested and Luciferase expression was measured by the chemiluminescence assay. Data obtained were plotted as 4-parameter logistic curve, and activation fold and EC_50_ were calculated:

(1) Activation fold=Chemiluminiscene in treated groupChemiluminiscene in control group.

### 2.4. Single Dose of E2HSA in Normal ICR Mice

#### 2.4.1. Oral Glucose Tolerance Test (OGTT)

Normal ICR mice were divided randomly into five groups (*n* = 10/group): normal saline treated group (Nor.), different dosages of E2HSA treated groups (1 mg/kg, 3 mg/kg, and 9 mg/kg resp.), and exendin-4 (2 *μ*g/kg) treated group. The doses of E2HSA were chosen based on its molecular weight, doses of similar large agonists of GLP-1R utilizing HSA [15, 20, 21], and preliminary experiments in our lab. Exendin-4 was used at the dose converted from the human equivalent dose (based on body surface area) [22] in the clinic (10 *μ*g, twice daily) to serve as a positive control. All mice were fasted overnight with water ad libitum before the experiment. E2HSA and exendin-4 were injected subcutaneously at doses described above and twenty minutes later all mice were orally challenged with glucose (2 g/kg). Blood samples were taken from the tail tip before E2HSA and exendin-4 injection (as 0 minute) and at 30, 60, and 120 minutes after glucose loading. To observe the lasting time of exendin-4, a second OGTT was carried out at about 4 h after the injection. On the 2nd day to 7th day of the injection, blood was sampled only at fasted state (as 0 minute) and at 30 min after glucose loading.

#### 2.4.2. Nonfasting Blood Glucose and Food Intake

Normal ICR mice were grouped, fasted, and treated as above. After injection, all mice were fed with normal chow. Blood glucose levels were then determined 1 h and 5 h later and daily on the 2nd day to 6th day. Food intake was recorded during the experiment, measured by weighing and calculating the differences of chow weight per cage between indicated time points. The sum was divided by sample size and then expressed as food intake per mouse within the indicated time period.

#### 2.4.3. Gastric Emptying Test

The experiment was staggered into four cohorts. Twenty mice in the first cohort were divided randomly into 2 groups (*n* = 10/group): normal saline treated group and exendin-4 (2 *μ*g/kg) treated group. The second cohort consisted of fifty mice which were divided randomly into five groups as described in Section 2.4.1. The third and fourth cohorts contained forty mice each and were both divided randomly into four groups as described in Section 2.4.1 but with subtraction of the exendin-4 treated group.

All mice were fasted overnight with water ad libitum. On the experiment day, mice were injected subcutaneously with E2HSA, exendin-4, or normal saline, respectively, at doses described above. Ink was orally given to different groups at 0.5 h (1st cohort), 5 h (2nd cohort), 24 h (3rd cohort), and 72 h (4th cohort) after the injection, respectively. Fifteen minutes later, mice were sacrificed and the small intestine was isolated; the total length of small intestine and the distance of ink delivered were measured. Gastric emptying rate was then calculated as the ratio of ink delivery distance/total length of small intestine.

### 2.5. Chronic Treatment of E2HSA in Spontaneous Type 2 Diabetes db/db Mice

#### 2.5.1. Animals Handling and Treatment

db/db mice were randomly divided into five groups: the vehicle (normal saline) treated group (Con.), three different dosages of E2HSA (1 mg/kg, 3 mg/kg, and 9 mg/kg, resp.) treated groups, and exendin-4 (2 *μ*g/kg) treated group. Ten age- and gender- matched db/m mice served as normal controls (Nor.). E2HSA was injected subcutaneously once daily at doses described above and exendin-4 was given twice daily at the dose of 2 *μ*g/kg. Both Con. and Nor. groups were injected with vehicle (normal saline) once daily. The treatment lasted for 43 days (about 6 weeks). At the end of study, all mice were decapitated and plasma samples were stored at −80°C for subsequent biochemical analysis. The pancreatic tissues were immediately removed and weighed. Samples were fixed for immunofluorescence analysis or stored at −80°C for subsequent preparation of total RNA and proteins.

#### 2.5.2. OGTT, IVGTT, and Plasma Biochemical Analysis

OGTTs were carried out weekly except for the 4th week. Fasting plasma glucose (FPG) levels and nonfasting plasma glucose (non-FPG) levels were measured every week, and, additionally, non-FPG levels at 1 hour and 5 hours after treatment on the first day were also measured. Nonfasting blood samples from 37th day were collected and HbA1c levels were evaluated using commercial kits (Beijing HOMA Biologicals, China). Fasting plasma insulin levels on the 20th day and 34th day were measured by ELISA (American Laboratories Product Co., USA.). For IVGTT, on day 40, after overnight food deprivation, 250 mg/kg glucose was injected through tail vein and blood samples were taken 2 min, 5 min, and 8 min after the intravenous glucose challenge; plasma insulin levels from each time point were analyzed. After the mice were sacrificed on 43rd day, blood samples were collected to test plasma glucagon levels using ELISA kit from R&D Systems, Inc., USA. Plasma triglyceride and total cholesterol levels were measured at the 1st week, 3rd week, and 5th week using commercial kits (BioSino Bio-technology and Science, Inc., China). Plasma FFA levels were evaluated at the 2nd week and 4th week also with commercial kits (Sekisui Medical, Tokyo, Japan). Food and water intake and body weights were monitored every day; the food and water data were then normalized to intake per mouse per week.

### 2.6. Immunofluorescence

After sacrifice, pancreases were dissected and fixed in aqueous Bouin's solution and then embedded in paraffin. Serial 5 *μ*m paraffin-embedded sections were mounted on slides. Two sets of stains were prepared. In one case, sections were dewaxed using xylene, rehydrated through serial dilutions of ethyl alcohol, and subjected to antigen retrieval using Tris-EDTA (pH 9.0). Sections were incubated overnight with a cocktail of two primary antibodies: rabbit anti-glucagon antibody (1 : 25) and rat anti-insulin antibody (1 : 45). The antigens were visualized using a cocktail of Fluorescein conjugated secondary antibody and Rhodamine conjugated secondary antibody (ZSGB-Bio, Inc., China). In another set, sections were labeled by TUNEL according to manufacturer's instructions followed by staining with rat anti-insulin antibody. All images were acquired through a fluorescence microscope equipped with a charge-coupled device camera (Olympus Inc. JPN). The ratio of insulin positive beta-cells to total islet area and the ratio of TUNEL positive *β*-cell to total insulin positive cells were calculated from digitized images captured under 20x objective using ImageJ software.

### 2.7. Western Blot

Total protein was extracted from frozen pancreas (the tail of pancreas) using RIPA lysis buffer (Applygen Technologies Inc., China) supplied with a protease and phosphatase inhibitor cocktail (Cell Signaling Technologies, USA). Protein was quantitated by BCA assay and denatured. Equal amounts of protein were resolved and separated electrophoretically by SDS-PAGE before transfer to polyvinylidene difluoride membranes. Membranes were then blocked for 1 hour with 5% nonfat milk in Tris-buffered saline (0.1% Tween-20) and different blots were incubated overnight with indicated primary antibodies (all in 1 : 500 dilution) at 4°C. After washing, blots were incubated at RT with horseradish peroxidase-conjugated secondary antibody (ZSGB-Bio, Inc., China). Proteins were visualized using an enhanced chemiluminescence detection system (ChemiScope 2850, Clinx Science Instruments, China) and band densities were analyzed by ImageJ software. The expression of *β*-actin protein served as internal standard. Data were expressed as fold expression relative to expression in vehicle treated Con. group.

### 2.8. Quantitative Real-Time PCR

Total RNA was extracted from frozen pancreas (the tail of pancreas) using TRIzol reagent (Invitrogen, USA) and further purified. Concentrations and 260/280 nm or 260/230 nm ratios of purified total RNA were analyzed by a Biodropsis BD-2000 spectrophotometer (OSTD Beijing Co. Ltd., China). cDNA was then synthesized using VigoScript First Strand cDNA Synthesis Kit (Vigorous Biotechnology Beijing Co., Ltd., China) and subjected to quantitative real-time PCR utilizing 7900 Real-Time PCR System (Applied Biosystems, USA). cDNA was amplified with SYBR Green Master Mix (Takara, China) using the following protocol: 1 cycle at 95°C for 30 s followed by 40 cycles at 95°C for 5 s and 60°C for 31 s. Specific PCR primers were designed by Primer-BLAST and synthesized by Invitrogen (Beijing, China). Data were calculated using delta-delta Ct (^ΔΔ^Ct) method and expressed as fold expression relative to expression in vehicle treated Con. group. The level of *β*-actin served as an internal standard.

Primer sequences used were as follows:

 ATGAACAGTGAGGAGCAGTAC′ and ACGGGTCCTCTTGTTTTCCTC for Pdx-1.
 CACAATTCCAAGCGCCACAA and CATCACCTCCTCCCAGGGTA for Irs2.
 CCATCAGCAAGCAGGAAGGTTA and GCTTGACAAAAGCCTGGGTG for Ins2.
 AGGCAAAGAGGGACATGCGGGA and TGCTTCAGCTCCTCT GCGGC for Igf1.
 CCGCGCCTCCCAACCTTGTT and TTCTCCA CCCCCGCGGGAAA for Nkx6.1.
 GCGCCTCAGGAAAAGCGGTG and AGCGCCTCGGGGTTCAGGTG for MafA.

### 2.9. Statistical Analysis

All values are presented as mean ± S.E.M. Data were analyzed by ANOVA followed by Bonferroni's correction and Student's *t*-test (two-tailed test). Analysis was performed using Excel (Microsoft, Redmond, WA) or Prism (GraphPad Software Inc., San Diego, CA). The *P* value less than 0.05 was considered to be statistically significant.

## 3. Results

### 3.1. GLP-1 Receptor Activation in NIT-1 Cells

**Figure 1:**
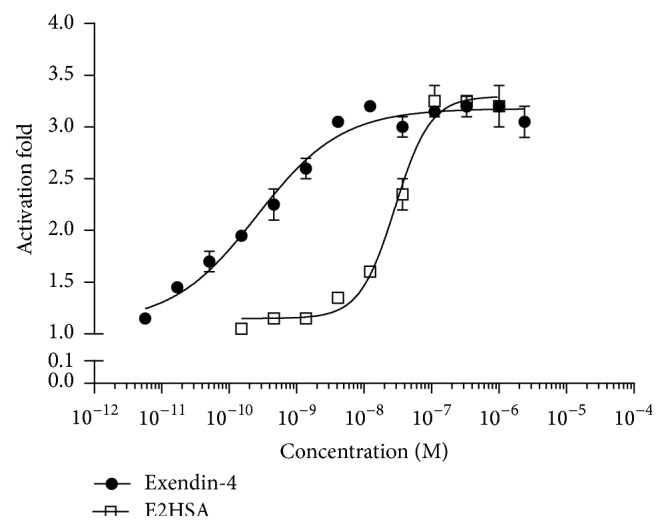
E2HSA exhibits GLP-1 receptor activating efficacy in NIT-1 cells. NIT-1 cells transiently transfected with Peak12 RIP-CRE 6x Luciferase reporter gene plasmid were treated with indicated concentrations of E2HSA and exendin-4 for 24 hours. Luciferase expression in cell lysates was measured by chemiluminescence. Data obtained were used to calculate the activation fold at different concentrations. Values are expressed as means ± S.E.M and are representative of data from two independent experiments, each performed in quadruplicate.

E2HSA produced a dose-dependent activation of the GLP-1 receptor in NIT-1 cells with concentrations ranging from 0.1 nM to 1000 nM. Compared to exendin-4, E2SHA showed a similar max GLP-1R activation fold (3.3-fold) but different EC_50_ (28.2 nM for E2HSA versus 0.215 nM for exendin-4) (Figure 1). The results showed that the recombinant fusion protein of exendin-4 and human serum albumin (HSA) possessed the same efficacy as exendin-4 to recognize and activate GLP-1 receptor but with lower potency perhaps due to steric hindrance of the HSA.

### 3.2. Extended Glucose Lowering and Gastric Emptying Effects after a Single Dose of E2HSA in Normal ICR Mice

**Figure 2:**
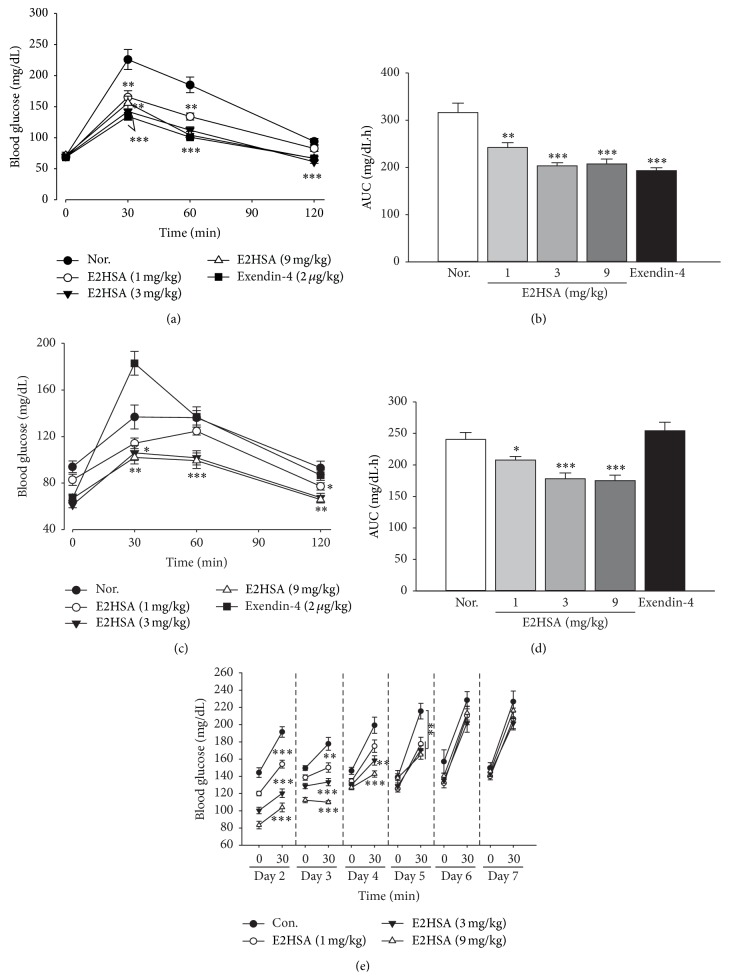
Long-acting, glucose lowering effect of E2HSA following oral glucose challenge in normal ICR mice administered a single dose. ((a)-(b)) Curves of blood glucose and AUC after first oral glucose loading performed at 20 minutes after administration of E2HSA. ((c)-(d)) Curves of blood glucose and AUC after second oral glucose challenge carried out at 4 hours after administration. (e) Blood glucose levels at fasting state and 30 minutes after oral glucose challenge on the 2nd day to 7th day after administration. Nor., normal ICR mice administered normal saline. Exendin-4, exendin-4 treated group. Data are expressed as mean ± S.E.M (*n* = 10). ^*∗*^ *P* < 0.05, ^*∗∗*^ *P* < 0.01, and ^*∗∗∗*^ *P* < 0.001 versus Nor.

**Figure 3:**
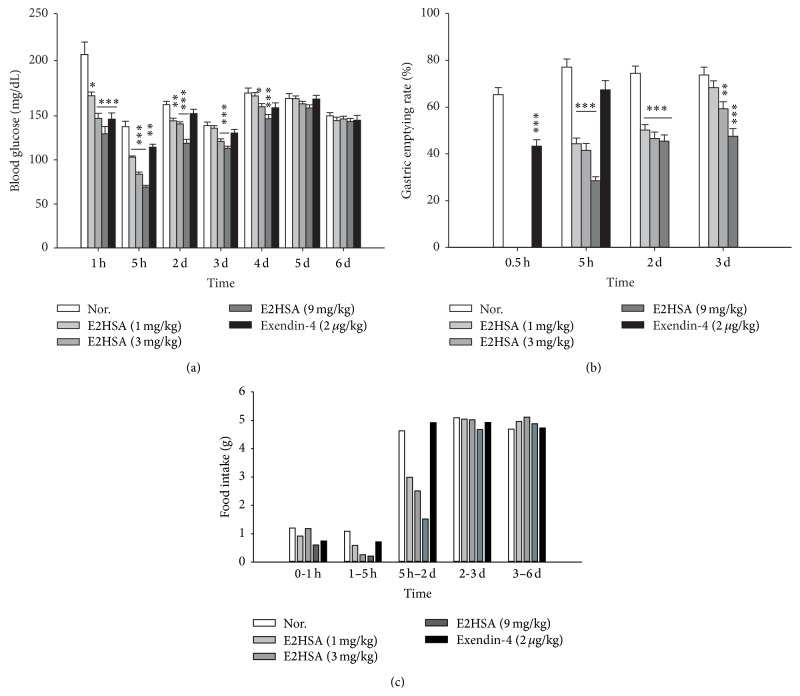
Long-acting effects of E2HSA on nonfasting blood glucose levels (a), gastric emptying (b), and food intake per mouse (c) in normal ICR mice injected with a single dose subcutaneously. Food intake was measured per cage and expressed as food intake per mouse within the indicated time period. Nor., normal ICR mice administered saline. Data are expressed as mean ± S.E.M (*n* = 10). ^*∗*^ *P* < 0.05, ^*∗∗*^ *P* < 0.01, and ^*∗∗∗*^ *P* < 0.001 versus Nor.

In normal ICR mice, a single administration of E2HSA dose-dependently reduced glucose levels and area under curves (AUC) after oral glucose challenge at 20 minutes and 4 hours after administration on the first day. On the other hand, exendin-4 (Ex-4) ceased to suppress elevated glucose levels at 4 hours after administration (Figures 2(a)–2(d)). Furthermore, from the second day to the fifth day, E2HSA still significantly suppressed the elevated blood glucose levels at 30 minutes after oral glucose challenge. Eventually, the effect of E2HSA on blood glucose levels diminished on the last two days (Figure 2(e)). Thus, the glucose lowering effect of E2HSA could last at least 4 days and in a dose-dependent manner. We also observed such changes in nonfasting blood glucose levels after a single administration of E2HSA (Figure 3(a)). As expected, E2HSA displayed an extended, dose-dependent blood glucose lowering effect that lasted to the 4th day, though the effect of 1 mg/kg E2HSA was not significant on the 3rd and 4th days. Notably, exendin-4 lost its effect on the second day.

To validate the effect of E2HSA on gastric emptying, we measured the delivered distance of orally administered ink in the small intestine and the total length of the small intestine to calculate the gastric emptying rate (Figure 3(b)). The rates in E2HSA-treated groups were significantly lower than those in saline-treated normal groups, suggesting that gastric emptying and small intestine peristalsis were inhibited. This effect was also dose-dependent and could last to the 3rd day after only a single administration of E2HSA. On the other hand, we could not observe any inhibition on gastric emptying 5 hours after administration in the exendin-4-treated groups. Consistent with its inhibition of gastric emptying, food intake in E2HSA-treated ICR mice also showed a reduction up to the 2nd day after a single administration (Figure 3(c)). One hour after administration, the effects of E2HSA and exendin-4 on food intake were comparable (dropped by 23.3% and 50% for 1 mg/kg and 9 mg/kg E2HSA, respectively, and by 38% for exendin-4). At 5 hours after administration, the reduction in food intake was 45.9%, 76.1%, and 80.7% for 1 mg/kg, 3 mg/kg, and 9 mg/kg E2HSA, respectively, while the reduction with exendin-4 administration remained at 34.9%. On the second day, exendin-4 no longer had any effect on food intake, but E2HSA could still decrease food intake by 35.4%, 45.7%, and 67.1%, respectively. The effect of E2HSA on food intake became subtle on the 3rd day and disappeared thereafter.

### 3.3. Chronic Treatment of E2HSA Improved Glycemic Control in db/db Mice

**Figure 4:**
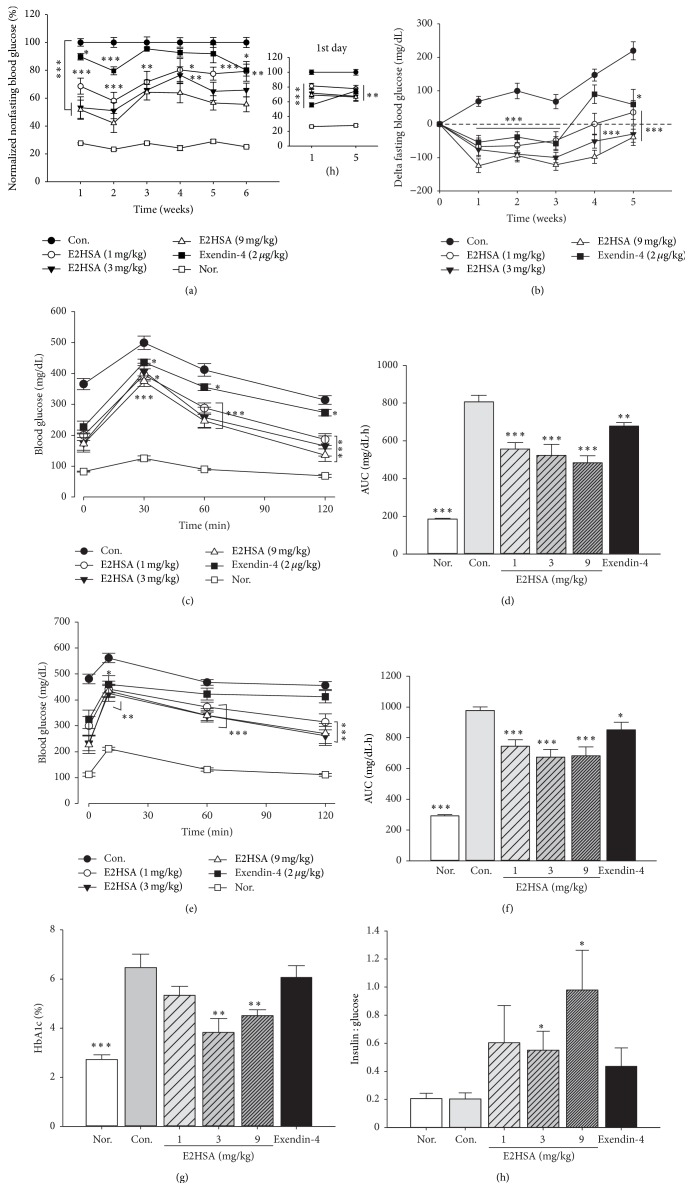
Chronic treatment with E2HSA improved glycemic control in spontaneous type 2 diabetes db/db mice. (a) Nonfasting blood glucose levels normalized by setting blood glucose levels in the control group to 100%. The mini figure shows the nonfasting blood glucose levels at 1 hour and 5 hours after E2HSA and exendin-4 administration on the first day. (b) Changes in fasting blood glucose levels relative to levels before E2HSA treatment. ((c) and (d)) Blood glucose curves and AUC of OGTT on the 2nd week. ((e) and (f)) Blood glucose curves and AUC of OGTT on the 5th week. (g) HbA1c levels on the 37th day. (h) Insulin/glucose ratios at 10 minutes following glucose challenge on the 5th week. Nor., db/m mice administered normal saline. Con., db/db mice administered normal saline. Data are expressed as mean ± S.E.M (*n* = 10-11). ^*∗*^ *P* < 0.05, ^*∗∗*^ *P* < 0.01, and ^*∗∗∗*^ *P* < 0.001 versus Con.

During 43 days of treatment, 1 mg/kg, 3 mg/kg, and 9 mg/kg doses of E2HSA all significantly decreased nonfasting and fasting blood glucose levels in a dose-dependent manner, and such effects were maintained throughout the entire treatment (Figures 4(a)-4(b)). Exendin-4 showed a comparable reduction in nonfasting and fasting blood glucose levels for the first two or three weeks, but then its efficacy became variable and the glycemic control in that group was worse than E2HSA-treated groups in the following weeks. OGTTs were performed on the 1st, 2nd, 3rd, and 5th weeks of E2HSA administration (the data from 1st and 3rd week are not shown). The results of the 2nd and 5th weeks are shown in Figures 4(c)–4(f). Glucose tolerance in E2HSA-treated db/db mice was significantly improved at all weeks tested. By the 2nd week, blood glucose levels following glucose challenge decreased significantly; the AUC in three doses of E2HSA-treated groups dropped 31.5%, 35.2%, and 40.1%, compared to the control group (Figures 4(c)-4(d)). By the 5th week, glucose levels after glucose challenge were still greatly reduced by all doses of E2HSA, with 23.8%, 31.0%, and 30.1% reduction in AUC, respectively (Figures 4(e)-4(f)). Ratios of insulin to glucose at 10 minutes after oral glucose loading were also increased significantly in groups treated with 3 mg/kg and 9 mg/kg E2HSA (Figure 4(h)), suggesting improved *β*-cell function. Exendin-4 significantly reduced the AUC (e.g., 16.0% and 13.0% at 2nd and 5th weeks, resp.) as well as suppressed blood glucose levels following glucose challenge, as shown in Figures 4(c)–4(f). Glycated hemoglobin, HbA1c, was tested at the end of E2HSA treatment. Compared to db/m mice, db/db mice in the control group displayed elevated HbA1c levels. On the 37th day of treatment, 3 mg/kg and 9 mg/kg E2HSA both reduced HbA1c levels significantly, indicating efficient glycemic control over the entire course of treatment (Figure 4(g)). On the other hand, exendin-4 was unable to show a significant HbA1c lowering effect at the same time (Figure 4(g)).

### 3.4. Long-Term Treatment of E2HSA Increased Insulin Secretion, Reduced Plasma Glucagon Levels, and Decreased the Plasma Lipid Profile

**Figure 5:**
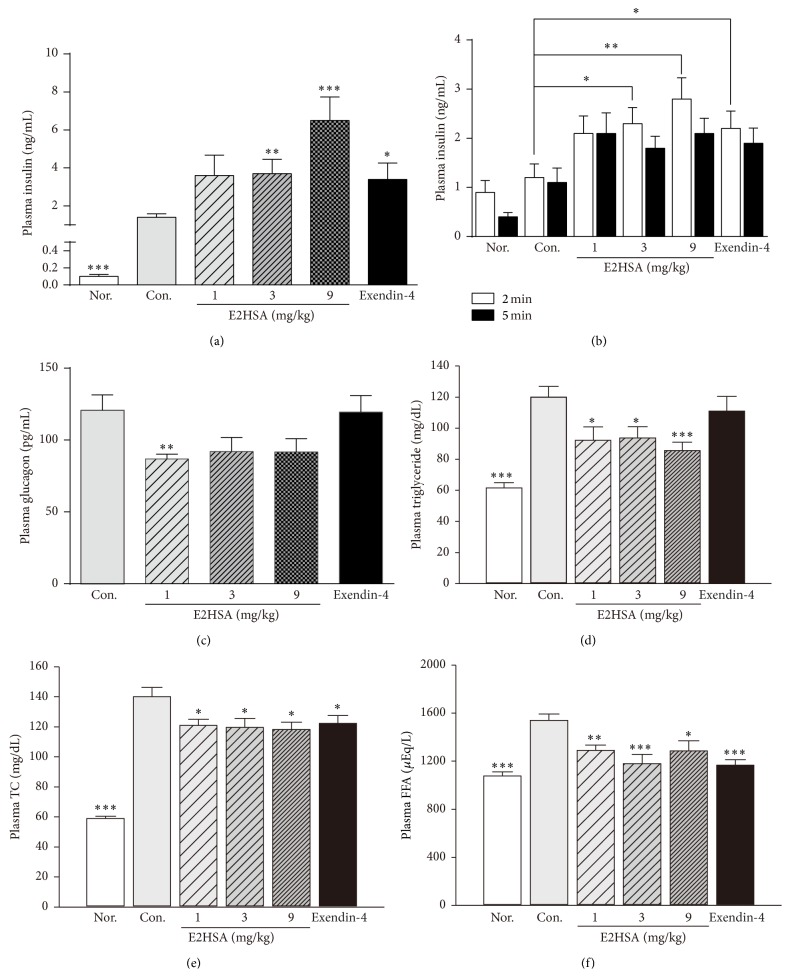
Effects of E2HSA on islet hormone secretion and plasma lipid levels in spontaneous type 2 diabetes db/db mice. (a) Fasting plasma insulin levels at the 2nd week. (b) Plasma insulin levels at 2 minutes and 5 minutes after intravenous glucose challenge in IVGTT. (c) Fasting plasma glucagon levels at the end of the experiment. ((d)–(f)) Fasting plasma triglyceride levels from the 5th week (d), fasting total plasma cholesterol levels from the 1st week (e), and fasting plasma FFA levels from the 2nd week (f) were represented here. Nor., db/m mice administered normal saline. Con., db/db mice administered normal saline. Data are expressed as mean ± S.E.M (*n* = 10-11). ^*∗*^ *P* < 0.05, ^*∗∗*^ *P* < 0.01, and ^*∗∗∗*^ *P* < 0.001 versus Con.

Chronic E2HSA treatment dose-dependently increased fasting plasma insulin levels in db/db mice (Figure 5(a)), while fasting plasma glucagon levels in E2HSA-treated groups showed an overall trend towards reduction (decreased by 28.0%, 23.7%, and 24.1% for 1 mg/kg, 3 mg/kg, and 9 mg/kg E2HSA, resp.) but with no dose-dependency (Figure 5(c)). Exendin-4-treated db/db mice displayed enhanced insulin secretion, while their plasma glucagon levels were comparable to the control group. To determine whether E2HSA treatment could increase phase I insulin secretion, we measured the acute insulin-secretory response to glucose by utilizing a simplified IVGTT. Blood samples taken at 2 minutes, 5 minutes, and 8 minutes after glucose challenge were analyzed. Insulin levels at 2 minutes were higher than those at 5 minutes and 8 minutes (insulin levels at 8 minutes were the lowest and not shown). Both 3 mg/kg and 9 mg/kg doses of E2HSA produced a significant increase in plasma insulin levels at 2 minutes (Figure 5(b)). Since first-phase insulin secretion occurs within the first 10 minutes after glucose infusion, we could conclude that chronic E2HSA and exendin-4 treatment significantly increased first-phase insulin secretion. During the treatment, E2HSA also significantly reduced fasting plasma triglyceride levels on the 1st week (not shown), 3rd week (not shown), and 5th week (Figure 5(d)) in db/db mice. Total plasma cholesterol and FFA levels were decreased in the first two weeks (Figures 5(e)-5(f)), but the effects were not sustained in the following weeks (data not shown).

### 3.5. Chronic E2HSA Treatment Decreased Body Weight, Food Intake, and Water Consumption

**Figure 6:**
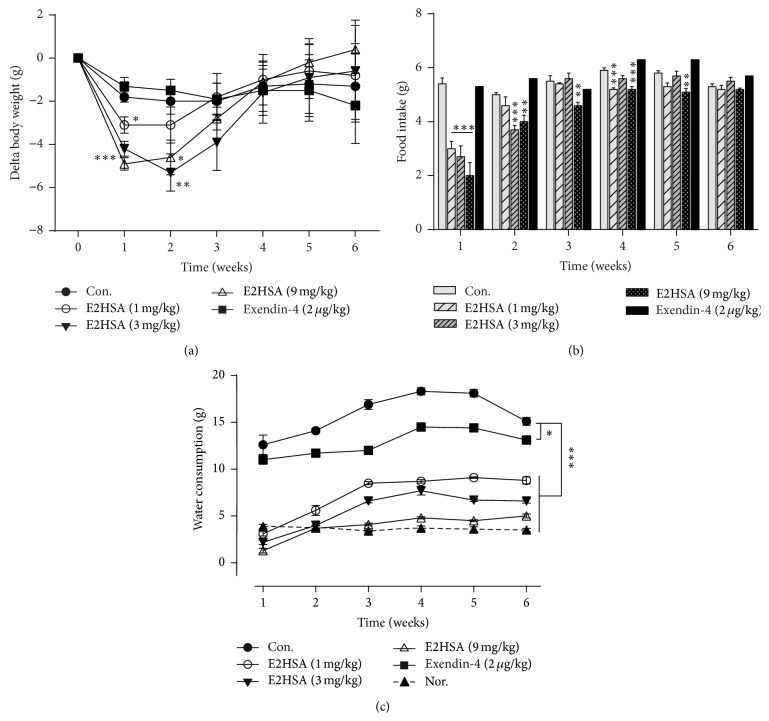
Chronic treatment with E2HSA decreased average body weight, inhibited average food, and water intake in spontaneous type 2 diabetes db/db mice. (a) Delta body weight change per mouse per week. (b) Average food intake per mouse per week. (c) Average water consumption per mouse per week. Con., db/db mice administered normal saline. Data are expressed as mean ± S.E.M (*n* = 10-11). ^*∗*^ *P* < 0.05, ^*∗∗*^ *P* < 0.01, and ^*∗∗∗*^ *P* < 0.001 versus Con.

During the chronic treatment of db/db mice, we monitored changes in body weight and food and water intake every day. As shown in Figure 6(a), E2HSA significantly decreased body weight in the first two weeks, although this effect diminished gradually. Body weight in the exendin-4-treated group showed only a very modest declining trend at the end of treatment. Food intake in all E2HSA-treated mice was significantly reduced in the first week. Interestingly, 9 mg/kg E2HSA maintained this effect until the 5th week, while the other two doses gradually lost efficacy (Figure 6(b)). On the other hand, Figure 6(c) showed that there was extensive water consumption in vehicle treated db/db mice compared to db/m mice, suggesting that diabetes-induced polydipsia might exist in these mice. E2HSA significantly reduced water consumption in a dose-dependent manner throughout the treatment (Figure 6(c)).

### 3.6. Chronic Treatment with E2HSA Restored *β*-Cell Morphology, Increased *β*-Cell Area, and Inhibited *β*-Cell Apoptosis

**Figure 7:**
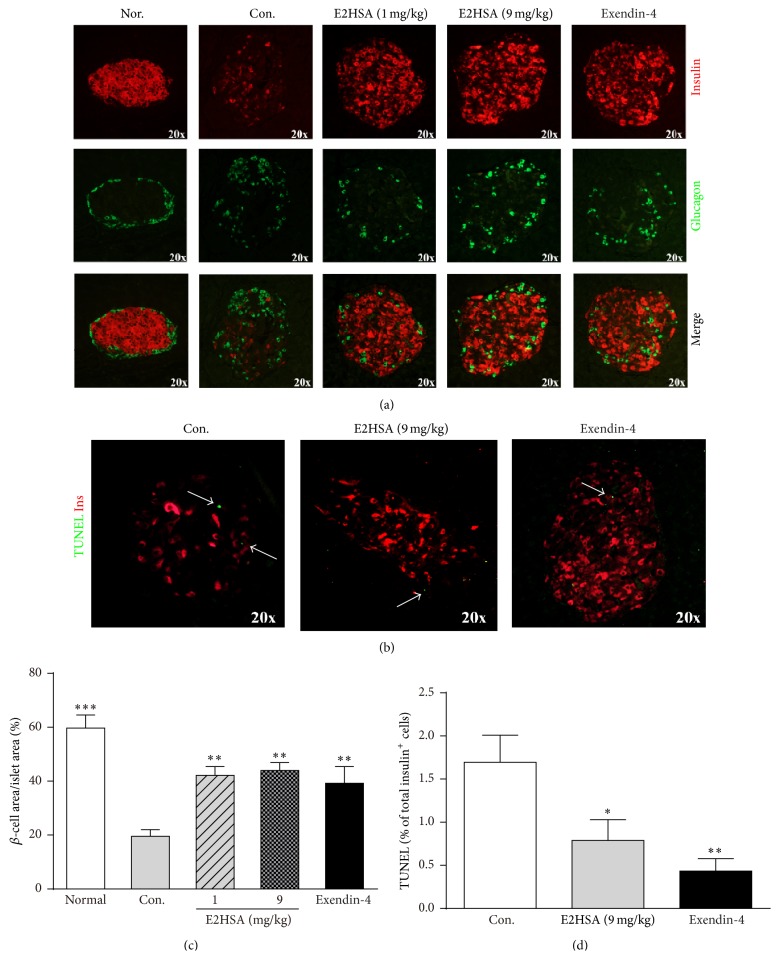
Chronic treatment with E2HSA normalized islet morphology, increased *β*-cell area, and inhibited *β*-cell apoptosis in spontaneous type 2 diabetes db/db mice. (a) Immunofluorescence double staining with anti-insulin and anti-glucagon antibodies on pancreatic sections. (b) TUNEL assay on *β*-cells costained with anti-insulin antibodies. Representative images are shown. (c) Total *β*-cell area as percentage of total islet areas (*n* = 5). (d) Percentage of TUNEL positive *β*-cells in insulin positive cells (*n* = 3 per group). Data are expressed as mean ± S.E.M. ^*∗*^ *P* < 0.05 and ^*∗∗*^ *P* < 0.01 versus Con.

Long-term treatment with E2HSA in db/db mice improved glucose tolerance and stimulated first-phase insulin secretion, suggesting an enhancement in *β*-cell function. To determine whether E2HSA had any effect on islet morphology and *β*-cell area, we double-stained islets with anti-insulin and anti-glucagon antibodies. As previously reported, islet morphology was impaired in db/db mice. Compared to db/m mice, *β*-cell area in db/db mice was less and the normal distribution of *α*-cells and *β*-cells was also perturbed. Intriguingly, E2HSA treatment significantly increased *β*-cell area and restored normal distribution of *α*-cells and *β*-cells, with *α*-cells on the outside and *β*-cells on the inside (Figures 7(a) and 7(c)). TUNEL assay revealed that chronic E2HSA treatment reduced the ratio of TUNEL positive nuclei to insulin positive *β*-cells, suggesting that *β*-cell apoptosis was also reduced (Figures 7(b) and 7(d)).

### 3.7. Chronic E2HSA Treatment Improved *β*-Cell Function and Survival That Correlates with the Regulation of Genes and Proteins Associated with Proliferation and Apoptosis

**Figure 8:**
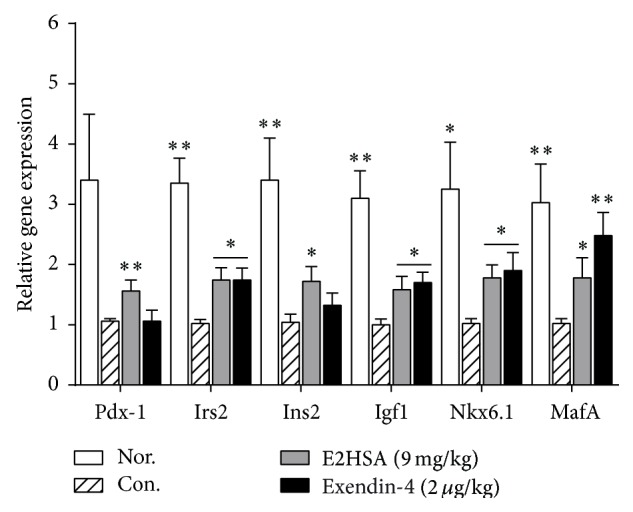
Expression of genes related to *β*-cell function and survival in spontaneous type 2 diabetes db/db mice treated with E2HSA. Data were from pancreas tail samples. Gene expression was analyzed by quantitative real-time PCR. A comparative cycle threshold (CT) method was used for relative quantification of gene expression between different groups using *β*-actin for normalization. Data are expressed as mean ± S.E.M (*n* = 4-5). ^*∗*^ *P* < 0.05 and ^*∗∗*^ *P* < 0.01 versus Con.

**Figure 9:**
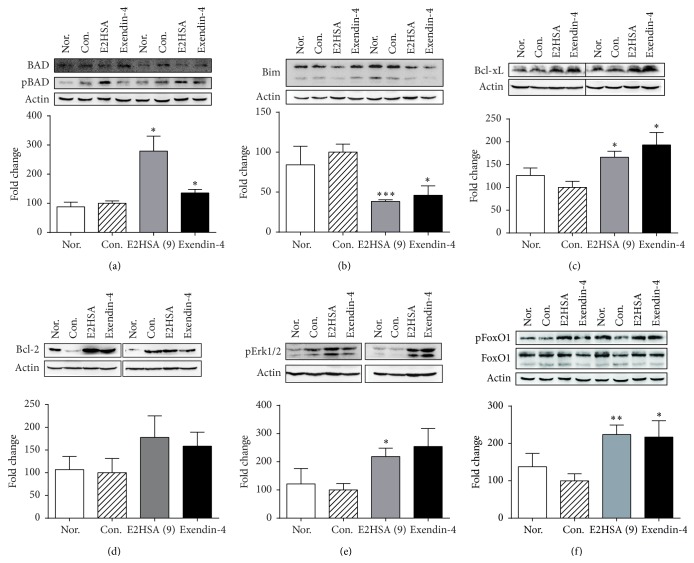
Chronic treatment with E2HSA regulated the expression of Bcl-2 family proteins and FoxO1. Data are from pancreas tail samples. ((a)–(f)) The upper panels show 2 representative Western blots from each group. Protein bands were determined by densitometry analysis and expressed as fold change, after correction for *β*-actin expression levels, relative to Con. mice. ((a), (f)) The ratio of phosphorylated protein to total protein of each group was first determined; then the fold change was calculated by comparing to Con. mice. Nor., db/m mice treated with normal saline. Con., db/db mice treated with normal saline. E2HSA (9), db/db mice treated with 9 mg/kg E2HSA. Data are expressed as mean ± S.E.M (*n* = 4-5). ^*∗*^ *P* < 0.05, ^*∗∗*^ *P* < 0.01, and ^*∗∗∗*^ *P* < 0.001 versus Con.

Since chronic E2HSA treatment significantly increased *β*-cell area in db/db mice, we next used quantitative real-time PCR and Western blot to investigate whether the expressions of genes and proteins associated with *β*-cell survival and apoptosis were affected by E2HSA using samples made from the pancreas tail section. As shown in Figure 8, expression of Irs2, an essential component of the GLP-1 and insulin signaling pathways, was increased by 1.8-fold and 1.7-fold in E2HSA (9 mg/kg) and exendin-4 treated groups, respectively. Another downstream target of GLP-1, Pdx-1, was also upregulated by E2HSA (9 mg/kg). Bcl-2 family proteins are critical to *β*-cell survival and linked to GLP-1R activation. The proapoptotic factors, BAD and Bim, interact with Bcl-XL/Bcl-2 and result in cell death, while phosphorylated BAD (pBAD) is the inactive form and thus correlates with less death. We have demonstrated that E2HSA (9 mg/kg) treatment significantly increased pBAD (Ser112)/BAD ratios and decreased Bim expression levels (Figures 9(a)-9(b)), whereas prosurvival Bcl-XL protein expression levels were significantly upregulated and expression of Bcl-2 displayed a tendency towards enhancement (Figures 9(c)-9(d)). At the same time, E2HSA treatment also promoted the phosphorylation of an upstream regulator of BAD, Erk1/2, which phosphorylates BAD at Ser112, thus further reducing proapoptotic signals (Figure 9(e)). FoxO1 plays an important role in *β*-cell apoptosis and phosphorylation of FoxO1 leads to its inactivation. After chronic treatment with E2HSA, the pFoxO1/FoxO1 ratio was greatly augmented in db/db mice islets, indicating its activity was inhibited (Figure 9(f)). In accordance with increased insulin secretion, the expression levels of two important genes involved in the regulation of insulin biosynthesis and secretion, Nkx6.1 and MafA, were both significantly increased by about 1.8-fold after E2HSA treatment (Figure 8). E2HSA also upregulated Ins2 and Igf1, two genes which are involved in insulin-induced signaling pathways.

## 4. Discussion

GLP-1 based therapies drew a lot of attention in recent years due to its preferable clinical efficacies and unique action mechanisms. GLP-1R agonists have the advantage of simultaneously stimulating insulin secretion while inhibiting gastric emptying, which eventually leads to effective blood glucose control and weight loss [6]. Therefore, GLP-1R agonists such as exendin-4 and liraglutide represent a promising drug class and have been recommended as the second-line therapy for T2DM patients [23]. However, exendin-4 is relatively short-lived in vivo. Therefore, long-acting GLP-1 receptor agonists which can extend circulating time and reduce injection frequency are highly sought after in the clinic.

E2HSA is a recombinant protein generated by the fusion of two tandem exendin-4 peptides with human serum albumin, a configuration which significantly increases the half-life of exendin-4. In the present study, E2HSA could activate GLP-1 receptor and displayed a prolonged biological acting time in vivo. Utilizing luciferase reporter gene expression assays, we demonstrated that E2HSA not only retained the ability of exendin-4 to activate GLP-1R but also showed the same efficacy as exendin-4, suggesting that altered peptide conformation did not prevent exendin-4 from recognizing and activating the GLP-1 receptor. For its long-acting effects, we observed the lasting time of its glucose lowering effect in vivo shown by suppression of blood glucose levels after oral glucose challenge and reduction in nonfasting blood glucose levels. Compared to exendin-4, the circulating time of E2HSA was much longer and, according to our study, the effect of E2HSA on blood glucose could last up to 4 days in ICR mice. The inhibition of gastric emptying could also last at least 3 days. Thus, with the fusion of HSA, the duration of E2HSA's biological activity in vivo was significantly prolonged. Moreover, our results were further supported by the study of Zhang and colleagues [24]. They demonstrated that, in healthy rhesus monkeys, the biological half-life of E2HSA was approximately 54 h following subcutaneous administration, whereas exendin-4 had a much shorter half-life of 60 min.

Zhang et al. also evaluated the acute effects of E2HSA in healthy monkeys. After a single injection, E2HSA reduced fasting and nonfasting blood glucose levels, improved glucose tolerance, and decreased food intake. Using a hyperglycemic clamp, E2HSA also augmented insulin secretion, indicating improved *β*-cell function. All aforementioned effects lasted significantly longer than those using unmodified exendin-4. In our study, chronic treatment with E2HSA in spontaneous db/db mice revealed similar effects. The glucose lowering effect of E2HSA was robust, while glucose tolerance and *β*-cell function were improved and ultimately food intake and body weight were both greatly reduced. In addition, we also found that E2HSA could reduce apoptosis and promote *β*-cell survival.

E2HSA possessed the pharmacological actions of a GLP-1R agonist shown by improved glycemic control as well as by a reduction in HbA1c levels in chronic treatment. While 3 m/kg and 9 mg/kg doses of E2HSA significantly reduced HbA1c levels, the effect was not as remarkable with 1 mg/kg dose of E2HSA and was not also as remarkable in exendin-4-treated groups. HbA1c reflects the average blood glucose levels in the previous 2-3 months [25]. As our HbA1c data were obtained on the 37th day, just over 1 month, such data might have reflected the glycemic conditions before E2HSA and exendin-4 administration. It is also worth noting that the nonfasting and fasting blood glucose levels in 1 mg/kg E2HSA and exendin-4-treated groups were more variable in the 3rd week to 5th week, so it is possible that the effect on HbA1c levels was less significant. In fact, a recent study has demonstrated that after 4 weeks of treatment with exendin-4 (24 nmol/kg), HbA1c levels were not reduced in high-fat diet-fed mice [15]. Moreover, in another study [26], after 12-13 weeks of treatment, exendin-4 (24 nmol/kg) significantly decreased HbA1c levels in db/db mice (C57BLKS/J-Lepr^db^/Lepr^db^). Therefore, it is possible that if 1 mg/kg E2HSA or exendin-4 was given for a longer time, we might be able to observe its obvious HbA1c lowering effects.

Consistent with other GLP-1R agonists [26, 27], E2HSA dose-dependently increased plasma insulin levels and first-phase insulin secretion. Fasting plasma glucagon levels were decreased to some extent after E2HSA treatment, while in exendin-4-treated groups, the change was negligible. Although in clinical studies all GLP-1R agonists could inhibit glucagon secretion to varying degrees, few have measured plasma glucagon levels in experimental animals. Baggio et al. [20] reported that Albugon, another long-acting GLP-1R agonist, did not reduce plasma glucagon levels in mice fasted overnight. The same was also seen for CJC1134-PC [15]. However, another study [28] demonstrated that exenatide reduced basal plasma glucagon levels during a 30 min intravenous infusion (2.6 *μ*g/h) period in diabetic obese Zucker rats. Therefore, the effect of E2HSA on glucagon secretion needs to be further investigated.

Treatments with GLP-1R agonists are also associated with weight loss, which can be partly attributed to their ability to delay gastric emptying and increase satiety. As for E2HSA, the inhibition of gastric emptying lasted to the 3rd day after a single administration in ICR mice, which was notably longer than that achieved with exendin-4. A single administration of E2HSA in healthy monkeys also decreased food intake for about 4 days [24]. Furthermore, with chronic treatment in db/db mice, E2HSA was able to reduce body weight in the first two weeks but had no effect in the following weeks. The reduction in food intake displayed a similar pattern. Among other long-acting GLP-1R agonists, HFD-fed mice treated for 4 weeks with CJC-1134-PC [15] lost weight, but another GLP-1-albumin conjugate, CJC-1131 [21], failed to reduce body weight during 4 weeks of administration in db/db mice. Considering the fact that HSA is too large to cross the blood-brain barrier, such results can be partly explained by the indirect activation of GLP-1R through vagal afferent pathways. Anti-E2HSA antibodies may also play a part since the titers were much higher in the last three weeks (data not shown). It is of note that water consumption was significantly reduced throughout the study. These results are in line with clinical observations that thirst and polydipsia are linked to blood glucose levels in diabetic subjects, and the reduction could be the result of lower plasma glucose levels or improved glucose tolerance. Thorkildsen and colleagues [29] observed that the GLP-1 receptor agonist, ZP10A, improved glucose tolerance and decreased water consumption in db/db mice but had no effect on body weight. However, it is also possible that the reduction in water consumption contributed to the body weight loss we observe, but we cannot be sure as no further investigations were carried out in our study. Perhaps, for example, mice in E2HSA-treated groups urinated less? In fact one study [30] has shown that urine volume was decreased by exendin-4 (1 nmol/kg) treatment. So the relationship between water consumption and body weight needs to be further investigated. In addition, we did not observe any significant weight reduction in exendin-4-treated db/db mice. Other studies have observed similar results. In db/db mice treated with exendin-4 (24 nmol/kg) for 13 weeks [26] or with NN2211 (liraglutide) twice daily for 15 days [27] there was no significant reduction in body weight with either treatment. However, in rats (3, 10, and 30 *μ*g/kg/day) and HFD C57BL/6J mice (10 and 30 *μ*g/kg/day) [31], chronic treatment with exendin-4 for 28 days reduced body weight. So the mechanisms behind such controversial and contradictory observations still need to be fully elucidated.

GLP-1R agonists exert their pharmacological effects through multiple signal transduction pathways. In this study, we found that inhibition of *β*-cell apoptosis by E2HSA correlates well with the modulation of Bcl-2 family proteins. Bcl-2 and Bcl-XL, which are prosurvival factors, were upregulated, while BH3-only proteins, such as the proapoptotic factors, BAD and Bim, were downregulated [32]. Other studies have also reported that GLP-1R activation reduced apoptosis via increased expression of Bcl-2 and Bcl-XL [33]. BH3-only proteins function as initial sensors of apoptotic signals that emanate from various cellular processes [32] and interact with core Bcl-2 family proteins to promote apoptosis. BAD can heterodimerize with Bcl-XL or Bcl-2, replacing BAX from Bcl-2/Bcl-XL, thus neutralizing their protective, prosurvival effects and promoting cell death [34]. Once phosphorylated, pBAD is sequestered in the cytosol and apoptosis is blocked [34]. Hyperglycemia/glucotoxic stress increased BAD protein expression in human and mouse pancreatic islets and caused *β*-cell death [35]. Bim also induces apoptosis and can also interact with both Bcl-2 and Bcl-XL to antagonize their antiapoptotic activity [36]. Bim was found to mediate *β*-cell apoptosis induced by chronic exposure to high glucose [37]. Ren et al. [38] demonstrated that a knock-down of Bim significantly reduced *β*-cell apoptosis, preserved *β*-cell mass, and restored normal glucose tolerance in Irs2^−/−^ mice. Such processes also involve FoxO1, a transcription factor which had been shown to contribute to apoptosis by increasing transcription of Bim [39]. Importantly, in our study, expression levels of Bim were decreased and levels of phosphorylated FoxO1 were augmented in E2HSA-treated groups. Additionally, Irs2 gene expression was upregulated. FoxO1 is also directly involved in the proliferative and antiapoptotic actions of GLP-1 in *β*-cells [40]. Exendin-4 failed to stimulate *β*-cell replication or expansion of islet mass in transgenic mice with constitutive expression of FoxO1 in the nucleus [39], where its transcriptional activity was constantly activated [41, 42].

In addition to Irs2, there was also a significant increase in Ins2 and Igf1 gene expression levels upon E2HSA treatment. Irs2 (insulin receptor substrate 2) mediates the effects of insulin and Igf1 on *β*-cell growth and function [43], and Irs2 expression was strongly induced in *β*-cells by exendin-4 [44]. Upregulation of Irs2, Ins2, and Igf1 confirmed the improvement of *β*-cell function after E2HSA treatment. Pdx-1 is a critical regulator of mature *β*-cell function [45] and an important target for GLP-1. Exendin-4 failed to stimulate *β*-cell proliferation or inhibit apoptosis in Pdx-1^−/−^ islets [46]. It is thought that GLP-1 regulates Pdx-1 expression through the Irs2 signaling pathway [47]. FoxO1 may also play an important role in this process as Kitamura and colleagues [48] reported that FoxO1 reversed *β*-cell dysfunction in Irs2^−/−^ mice through partial restoration of *β*-cell proliferation and increased expression of Pdx-1.

Additionally, in vitro studies have suggested that the *β*-cell-enriched transcription factor, Nkx6.1, is involved in the regulation of insulin biosynthesis and secretion as well as *β*-cell proliferation [49, 50]. Moreover, MafA is an important factor for *β*-cell maturation, glucose-stimulated insulin secretion, and *β*-cell function [51], and its expression was decreased in db/db mice [52] coupled with reduced insulin secretion. Therefore, these two genes also contributed to the beneficial pharmacological effects of E2HSA on *β*-cells. Furthermore, Quoyer et al. [53] demonstrated in *β*-cells that GLP-1 activates the ERK1/2 cascade which then ultimately phosphorylates BAD at Ser112 and that this process protects *β*-cells against apoptosis. In our study, we observed a significant increase in pBAD (Ser112)/BAD ratio and phosphorylated ERK1/2 after E2HSA treatment. Thus we suspect E2HSA might exert its antiapoptotic effect partly through the ERK1/2 pathway.

## 5. Conclusion

The current preclinical study demonstrated that the recombinant fusion protein of exendin-4 and human serum albumin (E2HSA) retained the ability of exendin-4 to activate GLP-1 receptor with similar efficacy. E2HSA also displayed a much longer glucose lowering effect and a longer gastric emptying inhibitory effect in vivo. Chronic treatment confirmed its beneficial effects on glycemic control and insulin secretion as well as *β*-cell function and *β*-cell area. Importantly, *β*-cell apoptosis was also reduced and *β*-cell survival was promoted. Our data suggests that this novel, long-acting GLP-1R agonist possesses high antidiabetic potency and supports further assessment for once weekly treatment in T2DM patients.
